# The Formation of NETs and Their Mechanism of Promoting Tumor Metastasis

**DOI:** 10.1155/2023/7022337

**Published:** 2023-03-11

**Authors:** Jian Li, Jing Chen, Jing Sun, Kaichun Li

**Affiliations:** Department of Oncology, Shanghai Fourth People's Hospital, Tongji University School of Medicine, Shanghai 200434, China

## Abstract

Neutrophil extracellular traps (NETs) are network structures comprised of decondensed DNA strands coated with granule proteins. There have been three types of NETs recorded. NETs have been discovered concerning the progression of some malignancies, including gastric cancer, breast cancer, ovarian cancer, hepatocellular carcinoma, colorectal cancer, glioblastoma, diffuse large B cell lymphoma (DLBCL), and lung cancer, among others. In various methods, tumors encourage the formation of NETs, and NETs, in turn, promote tumor growth. NETs can stimulate primary tumor cell proliferation, suppress immune cells to create a tumor-friendly immune microenvironment, and stimulate epithelial-mesenchymal transition (EMT). NETs significantly promote liver and lung metastasis, possibly by altering vascular permeability, inducing cytoskeleton rearrangement and directional cell migration, and reawakening dormant cancer cells. NETs are therapeutically promising targets for cancer patients. Cancer patients may benefit from anti-NETs therapy, especially when combined with immune checkpoint inhibitors.

## 1. What Were NETs?

Neutrophil extracellular traps (NETs) were identified by Brinkmann [1] et al. in 2004 as network structure composed of decondensed DNA strands with diameters of 15–17 nm associated with bactericidal proteases produced by neutrophils in response to phorbol 12-myristate 13-acetate (PMA) and interleukin-8 (IL-8). NETs contain approximately 20 proteins, including neutrophil elastase (NE), myeloperoxidase (MPO), high mobility group protein B1 (HMGB1), peptidoglycan-binding protein, lactoferrin, pentraxin 3, cathepsin G, proteinase 3 (PR3), and cathelicidin (LL 37). NETosis is the process of NET formation, which was once believed to necessitate neutrophil death [2, 3]. Multiple microbial stimuli and proinflammatory mediators, such as bacteria, PMA [4], interleukins, IL-8, and IL-1*β*, induce NETosis. Cancer patients may benefit from anti-NETs therapy.

## 2. Two Main Forms of NETs Release Have Been Proposed

To date, two main forms of NETs release have been reported [2, 3, 5–11], namely, lytic NETosis or suicidal NETosis and nonlytic NET formation or vital NET formation [6, 12–15].

Suicidal NETosis is a nicotinamide adenine dinucleotide phosphate (NADPH) oxidase-dependent death process characterized by nuclear and granular membrane disintegration, chromatin decondensation, and the release of chromatin decorated with granular proteins and cell rupture. In this classical mechanism, neutrophils enter a cell death program that culminates with the release of NETs 1–4 hours after activation. Suicidal NETosis can be triggered by bacteria, fungi, viruses, antibody-antigen complexes, autoantibodies, concanavalin, and interferon [16, 17].

Different groups of investigators have described a “vital” form of NETs formation, in which the intracellular content is released in the extracellular space but cytoplasmic membrane rupture is not required and the neutrophil remains alive [18, 19]. Vital NETs release was first described under the granulocyte-macrophage colony-stimulating factor (GM-CSF) priming with subsequent stimulation with C5a or LPS [14]. Researchers [15] observed that under conditions of sepsis, platelets can induce the rapid release of NETs from neutrophils within minutes after the cell death. Vital NETs release was also observed after exposure to *Staphylococcus aureus* for 5 minutes [6] and 5–60 minutes [12]. Vital NETs take less time to form than suicidal NETs stimulated by PMA [17]. There are studies that have found vital NETs composed of nuclear DNA [12, 13]. In one study, NETs were released through blebbing of the nuclear envelope and vesicular exportation as opposed to plasma membrane rupture and lytic cellular death [12]. This entire process occurred via a unique, very rapid (5–60 minutes), oxidant-independent mechanism. In another study, polymorphonuclear cells *in vivo* rapidly release extracellular traps during Gram-positive bacterial skin infection [13]. During migration, normal cells utilize structurally stable nuclei as the fulcrum. In contrast, polymorphonuclear cells without a stable fulcrum crawl rapidly via unstable pseudopodia in a hyperpolarized state while retaining their phagocytic function. Some polymorphonuclear cells degenerated into non-nucleated cells that can still survive and maintain their bactericidal function. The nucleus seems to be the major source of NETs in this study.

In addition to vital NETs formation with nuclear DNA release, NETs composed of mitochondrial DNA were observed [6, 14, 20–23]. In 2009, Yousefi et al. proposed the formation of mitochondrial NETs from mitochondrial DNA [14]. They pretreated neutrophils with GM-CSF and then stimulated them with LPS or complement factor 5 a (C5a). After 15 to 20 minutes, a network of mitochondrial DNA rather than nuclear DNA was observed. Mitochondrial NETs were detected in the blood of patients with skeletal injuries and surgical operations [20] using quantitative polymerase chain reaction and a dye that is dependent on mitochondrial superoxide [24] and has a high affinity for mtDNA in the extracellular environment. In patients with anaplastic thyroid cancer (ATC), NETs induced by an ATC-conditioned medium contained mitochondrial DNA and promoted cancer cell proliferation [25]. Some studies [12] demonstrated that *Staphylococcus aureus* primarily induced Suicidal NETosis in the formation of NETs. Mitochondrial DNA exists in NET-DNA, although the proportion is less than 1 part in 100,000. Dunham−Snary et al. demonstrated that mitochondria are ejected from intact neutrophils and engage bacteria during vital NETs formation [6].

## 3. Many Factors Affect the Formation of NETs

The mechanism named suicidal NETosis has been shown to be dependent on reactive oxygen species (ROS) [26] generation by NADPH oxidase (NOX) and also chromatin decondensation dependent upon the enzymes (PAD4), neutrophil elastase (NE), and myeloperoxidase (MPO) [27–29]. Peptidyl arginine deiminase 4 (PAD4) is a calcium-dependent [11] enzyme in the nucleus that promotes the citrullination of histones H3, H2A, and H4. Citrullination of histones by PAD4 results in the loss of positive charge of histones and compactness of chromatin [30, 31]. The ensuing unwrapping of nucleosome is central events in the *in vivo* formation of NETs. Inhibition of PAD4 can substantially reduce histone citrullination and histone depolymerization, resulting in dysgenesis of NETs [29, 32].

Vital NETs release observed after exposure to *Staphylococcus aureus* for about 5 minutes was independent of ROS production by NADPH oxidases (NOX) [12] but dependent on mitochondrial complex III [6]. The vital NET formation is mediated by a TLR2-dependent mechanism independent of oxidants [13]. Researchers [15] observed that under conditions of sepsis, platelets can induce the rapid release of NETs from neutrophils within minutes through TLR4, enabling the capture of bacteria. It has been reported that the release process of NETs composed of mitochondrial DNA binding granular proteins requires the production of glycolytic ATP to rearrange the microtubule network and F-actin [33]. Douda et al. described a rapid NOX-independent NET formation process that is mediated by mitochondrial reactive oxygen species (ROS) and a calcium-activated small conductance potassium channel [34].

Tumor cells stimulate the production of neutrophils and NETs in the peripheral blood of cancer patients by secreting granulocyte colony-stimulating factor G-CSF [35, 36]. In a murine model of metastatic breast cancer, it was discovered that blocking the interleukin-1 receptor (IL-1R) can reduce the systemic G-CSF level and NETs production but does not affect neutrophil counts [35]. IL-8 immunostaining demonstrates a positive correlation with NETs [37]. Tumor-derived IL-8 [38], the most abundantly expressed C-X-C motif chemokine receptor 2 (CXCR2) ligand [37], can induce NETosis in a variety of cancers by interacting with the CXCR2 receptor on neutrophils [39]. Signaling of IL-8 to CXCR2 initiates the PI3K/AKT/reactive oxygen species (ROS) signaling cascade essential for NETs production.

GSDMD also plays an essential function in NETosis [40, 41]. GSDMD is a pore-forming protein and an executor of pyroptosis. Using high-resolution total internal reflection fluorescence (TIRF) microscopy, researchers found that GSDMD is cleaved during NET formation and then localizes to the plasma membrane of neutrophils. GSDMD is required for NETosis and affects nuclear expansion during NET formation.

## 4. Tumors Promote NETosis Generation

Neutrophils from cancer patients are more capable of producing NETs than those from healthy counterparts of comparable age. The patients with advanced esophageal, gastric, and lung cancer have elevated circulating NETs levels compared to healthy controls [42]. Coculturing neutrophils induced NETs formation with cancer cells in conditioned media. MPO-DNA can be indicative of NETs levels. The prognosis is poor for patients with elevated MPO-DNA in samples and serum, such as colorectal cancer patients with liver metastases [43] and patients with diffuse large B-cell lymphoma [44]. The study also revealed that the number of NETs was associated with the rapid growth of tumors and that a decrease hindered the generation of NETs in the number of mitochondria [43]. This section focuses primarily on the role of NETs in tumor progression.

The migration rate and the number of neutrophils in mice and humans exposed to a hypoxic medium are greater than that in those exposed to a normoxic medium. Hypoxia, which is frequently present in rapidly growing tumors, may attract neutrophils and cause the formation of NETs by upregulating HMGB1. HMGB1 levels increased significantly in the culture medium of human and mouse colorectal cancer and hepatoma cell lines treated with hypoxia for 24 hours. The addition of an HMGB1-neutralizing monoclonal antibody to a hypoxic cancer medium significantly inhibited the formation of NETs [45]. HMGB1 was discovered to bind the Receptor of Advanced Glycation Endproducts (RAGE) receptor on glioma cells *in vitro*, resulting in the activation of NF-*κ*B and subsequent upregulation of IL-8 expression, thereby promoting tumor progression in breast cancer.

NETs can be identified by colocalization of NE and H2B, as in intact neutrophils, both proteins reside in different cellular compartments, namely, NE in the cytoplasmic granules and H2B in the nucleus [46]. Other studies have employed H3cit as a marker of NETs in tissues, either alone or in conjunction with other markers [37, 47, 48].

## 5. NETs Promote Tumor Cell Proliferation

NETs have been found to promote tumor progression by increasing tumor cell proliferation in different kinds of tumors, such as murine colorectal cancer [43], glioblastoma [49], and DLBCL [50]. Higher expression of activating markers on leukemic B cells in the presence of NETs has been found [51]. Transplanted tumors in PAD4-deficient mice unable to form NETs showed delayed progression [52]. High mobility group box protein 1 (HMGB1) is a danger-associated molecular pattern (DAMP) protein. In a toll-like receptor 2 (TLR2) [53, 54] or toll-like receptor 4 (TLR4) [54–56]-dependent manner, HMGB1 is abnormally released into the tumor microenvironment (TME) and activates an inflammatory response that promotes tumor progression.

The hypoxic environment generated in the center of the hepatocellular carcinoma (HCC) causes the release of damage-associated molecular pattern (DAMP) proteins, including High Mobility Group Box 1 (HMGB1) and mitochondrial DNA (mtDNA). Liu et al. [57] discovered that HCC cells treated with a TLR9 agonist displayed a time-dependent increase in cell proliferation compared to the vehicle control. TLR9 is a DNA receptor widely expressed in various cancers and promotes tumor growth by activating intracellular growth signaling pathways [57–59]. By silencing HMGB1 with shRNA or removing mtDNA from cancer cells, the activation of p38, p65, JNK, and IL-6 expression could be significantly reduced. Depleting HMGB1 and mtDNA in the same cell or adding a TLR9 antagonist led to a more pronounced reduction in MAP kinase activation.

Moreover, the outcomes of MAP kinase activation in the various groups paralleled the outcomes of tumor cell proliferation. The JNK family and p38 isoforms that are highly activated by environmental stresses and inflammatory cytokines can contribute to the proliferation of cancer cells. In response to stress, NF-*κ*B signaling can suppress apoptosis and the continuation of cell proliferate. Mitogen-activated protein (MAP) kinase pathways regulate fundamental cellular processes, including growth, proliferation, differentiation, and migration, which are all essential aspects of tumor development. Studies demonstrate that the tumorigenic effects of TLR9 are dependent on NF-*κ*B-mediated upregulation of IL-6 expression [60]. Extracellular HMGB1 can stimulate cancer invasion and metastasis via Toll-like receptor (TLR)-4 signaling [61] and promotes the formation of NETs *in vitro* in a TLR-dependent manner. The inhibition of extracellular HMGB1 and NETs *in vivo* significantly retards the growth of bladder tumors [62].

Signaling between PDGFR and PI3K is required for NE-induced proliferation [50]. Neutrophil elastase directly induced tumor cell proliferation in human and mouse lung adenocarcinomas by entering an endosomal compartment and degraded insulin receptor substrate-1 (IRS-1). Increased interaction between phosphatidylinositol 3-kinase (PI3K) and the potent mitogen platelet-derived growth factor receptor (PDGFR) shifted the PI3K axis towards tumor cell proliferation.

NETs regulate mitochondrial homeostasis to promote tumor growth. NETs are associated with a pro-proliferative active metabolic response and preservation of mitochondrial function, and mitochondrial homeostasis co-occurs with increased energy production in tumors and accelerated tumor growth. Neutrophil elastase secreted by NETs stimulates mitochondrial biogenesis via the TLR4-p38-PGC-1*α* pathway [43]. *In vitro*, cancer cells treated with NETs increased mitochondrial biogenesis-related genes, mitochondrial density, and ATP production. Mitochondrial biogenesis induced by peroxisomes proliferator-activated receptor gamma coactivator 1-alpha (PGC1-*α*) results in an increase in cellular energy that is conducive to anabolic tumor growth.

When cancer cells were treated with CTSC- or PMA-induced NET media in a three-dimensional culture system, tumor spheroids grew at an accelerated rate [63].

## 6. Impact of NETs on the Immune Microenvironment

Time-lapse confocal microscopy and image quantification demonstrated that CD8+ T cell and NK cell contacts with tumor cells were significantly diminished in the presence of NETs surrounding the tumor cells [39]. NETs in mice injected with Lewis lung carcinoma (LLC) cells decrease the physical contact between cancer cells and cytotoxic lymphocytes [39]. NETs coat and shield tumor cells from cytotoxicity are mediated by NK and T cells. CXCR1 and CXCR2 receptors are expressed on neutrophils and granulocytic myeloid-derived suppressor cells (GR-MDSCs) [38]. Chemokines that act through the CXCR1 and CXCR2 chemokine receptors can induce NETosis on neutrophils. Reparixin is an allosteric inhibitor of CXCR1 and CXCR2 that inhibits NETs extrusion as induced by CXCR1 and CXCR2 agonists. Blocking CXCR1 and CXCR2 with Reparixin or a CXCR1 blocking monoclonal antibody (mAb) inhibited conditioned-supernatant-induced NETosis completely [39]. The combination of the CXCR2 inhibitor SX-682 and anti-PD-1 significantly prolonged the survival of mice with colorectal cancer, whereas anti-PD-1 alone did not [64]. Genetic ablation of host CXCR2 prevented neutrophil accumulation in pancreatic tumors and led to T cell-dependent tumor growth suppression [65]. Migration of CD8+T cells to CCL5 was severely inhibited, but DNase-I-mediated removal of NETs restored migration of CD8+T cells to CCL5 [39]. Even without tumor cells, NETs inhibited lymphocyte motility [39].

The loss of T cell function in the tumor microenvironment (TME) is caused by an environment rich in NETs. The density of CD8+ T lymphocytes correlated negatively with the size of NETs in NSCLC and bladder cancer [37]. In a model of colorectal liver metastases, T cells in a murine tumor environment rich in NETs display a phenotype of T cell exhaustion and dysfunction [66]. In this study, T cells cultured with mouse NETs expressed significantly higher levels of exhaustion markers (PD-1, Tim3, Lag3), produced less cytokine, had fewer mitochondria, and exhibited impaired mitochondrial function and glucose uptake. After stimulation *in vitro* with CD3/28 beads, the proliferative capacity of these T cells was also diminished. The phenomenon mentioned above was also observed *in vitro* when T cells were exposed to NETs but not PD-L1-deficient NETs. In this study, NETs were also found to be the primary source of PD-L1 in livers with NETs-rich TME, as determined by flow cytometry with staining for NETs and PD-L1. The results, as mentioned earlier, demonstrated that the PD-L1 found in NETs is responsible for the *in vitro* exhaustion of T cells. The treatment with anti-PD-L1 diminished the tumor size and the number of exhausted T cells in the tumor microenvironment (TME).

Regulatory T cells (Tregs) promote tumor cell survival by producing an immunosuppressive environment. Eliminating Tregs inhibits nonalcoholic steatohepatitis-associated hepatocellular carcinoma development (NASH-HCC) significantly. By enhancing mitochondrial respiratory function, NETs modulate the regulatory gene profiles of naive CD4+T cells and promote their differentiation into Tregs [67]. Toll-like receptor 4 is required for the metabolic reprogramming of naive CD4+T cells to promote Treg differentiation. The increase in T regulatory cells inhibited CD8+ T cell infiltration [67]. The low-density neutrophils (LDN) can produce NETs that efficiently trap gastric cancer cells *in vitro*, significantly inhibiting the proliferation of autologous T cells and partially inhibiting the cytotoxicity of lymphocytes against a human gastric cancer cell [68].

Multiple strategies that inhibit NETosis significantly inhibit spontaneous lung and liver metastasis. After intrasplenic injection, intravital microscopy revealed a significant increase in hepatic adhesion in tumor-bearing mice (TBM) compared to non-TBM and NET-deficient mice [42].

## 7. NETs Promote Epithelial-Mesenchymal Transition (EMT)

As we all known, there are two types of neutrophils, anti-tumourigenic N1 phenotype and pro-tumourigenic N2 phenotype, although no specific marker could be used to differentiate these two subgroups. Jin W and colleagues discovered that neutrophils from pancreatic cancer patients alone promote the migration and invasion of cancer cells, whereas neutrophils from healthy individuals cannot. However, NETs induced from neutrophils of healthy donors and pancreatic cancer patients could promote the migration and invasion of pancreatic cancer cells. Following the loss of the epithelial phenotype and the acquisition of mesenchymal features, tumor cells acquire a potent ability to migrate and invade, resulting in distant metastasis. It has been demonstrated that invasiveness is associated with EMT. Through the IL1/EGFR/ERK pathway, NETs promote migration, invasion, and EMT in pancreatic ductal adenocarcinoma [69]. NETs were most prevalent in the tumor core and invasive front of colon cancer [70]. NETs were associated with decreased expression of epithelial markers E-cadherin (CDH1) [70, 71] and its mRNAs [69] and epithelial cell adhesion molecule (EPCAM) [70] and elevated levels of mesenchymal markers vimentin [71] and its mRNAs [69, 70], fibronectin [70], snail, and N-cadherin [69]. NETs also contributed to the increase of transcription factors that promote epithelial-mesenchymal transition (ZEB1, Slug [SNAI2]) [70]. The relationship between NETs and the expression of epithelial and mesenchymal markers have been identified in pancreatic cancer [69], colon cancer [70], and gastric cancer [71].

## 8. NETs Promote Tumor Cell Metastasis

Increased NETs deposition was associated with colorectal cancer liver metastasis [72]. Neutrophils cocultured with metastatic 4T1 cells generated extensive NETs, whereas neutrophils cocultured similarly with nonmetastatic 4T07 cells generated few NETs in breast cancer [73]. NETs induced by surgical stress or inflammation facilitate the metastatic seeding and colonization of tumor cells in host organs [48, 74]. The NET inhibitor prevents pancreatic cancer liver metastasis and the recruitment of activated cancer-associated fibroblasts (CAFs), a major component of the tumor microenvironment [75]. NETs were found in the omentum prior to metastasis in murine models of ovarian cancer and patients with early-stage ovarian cancer patients [76]. All of these findings suggest that NETs play a role in metastatic disease and that NETs may contribute to the formation of permissive metastatic niches [63].

Stimulation of endothelial cells with NETs resulted in morphological changes, including retraction of cell-cell junctions and a more procoagulative phenotype, which were reversed by DNase I [77] and activated protein C [78]. Changes in the morphology of endothelial cells and an increase in vascular permeability can increase cancer extravasation and promote tumor metastasis, such as breast cancer metastasis to the lungs [79].

NETs entrapped with colorectal cancer cells in the liver exerted no cytotoxicity but increased tumoral proliferation and invasion capability [72]. Tumor cells of murine Lewis lung carcinoma and human lung adenocarcinoma were physically entrapped by NETs [80]. Systemic sepsis encourages the development of gross metastasis, mitigated by systemic administration of inhibitors of NET formation. In a study [80], scanning electron microscopy revealed that NETs were wrapped around the adherent human cancer cells of the lungs and observed in direct contact with the tumor cell membrane.

Cathepsin C (CTSC), also known as dipeptidyl peptidase I, is required for the catalytic activation of numerous serine proteases, such as proteinase 3 (PR3), neutrophil elastase (NE), cathepsin G (CTSG), granzymes A/B/C, and mast cell chymase. By regulating neutrophil infiltration and NET formation in early metastatic niches, tumor-derived CTSC promoted the lung metastasis of breast cancer. CTSC induces the formation of NETs by activating p38 and promoting neutrophil production of reactive oxygen species. The NETs inhibitor Sivelestat or DNase I effectively reduced metastatic seeding and colonization of cancer cells in the lungs and diminished the effect of CTSC, thereby inhibiting lung metastasis [63]. This study confirmed that NETs degrade the metastasis-suppressing extracellular matrix (ECM) protein TSP-1 and promote lung metastasis.

CCDC25, upon sensing NET-DNA at AA21–25 on its extracellular domain, recruits integrin-linked kinase (ILK) via its intracellular C terminus and initiates the *β*-parvin-RAC1–CDC42 cascade to induce cytoskeleton rearrangement and directional migration of tumour cells [47].

The DNase I-coated nanoparticles did not affect the growth of the primary tumor but reduced the lung metastatic burden [73]. Fifty percent of mice treated with DNase I-coated nanoparticles had no histologically detectable micrometastases, whereas all mice treated with control nanoparticles had micrometastases [73].

## 9. NETs Awaken Dormant Cancer Cells

Cancer cells that can disseminate from a primary tumor to other tissues can lie dormant and be clinically undetectable for many years. Albrengues et al. discovered that persistent lung inflammation caused by exposure to tobacco smoke or nasal instillation of lipopolysaccharide converted disseminated, dormant cancer cells into aggressively growing metastases [48]. In this process, two NETs-associated proteases, neutrophil elastase, and matrix metalloproteinase 9, remodeled laminin and activated integrin *α*3*β*1 signaling, thereby awakening dormant cancer cells. Integrin *β*1 signaling plays a crucial role in the reawakening of cancer cells that have been dormant [81–83].

## 10. Conclusion

NETs may serve as diagnostic and prognostic biomarkers for cancer patients. NETs can promote primary tumor cell proliferation, inhibit immune cells to create a tumor-friendly immune microenvironment, and promote epithelial-mesenchymal transition (EMT). NETs significantly promote liver and lung tumor metastasis, possibly by altering vascular permeability, inducing cytoskeleton rearrangement and directional cell migration, and reawakening dormant cancer cells. Consequently, NETs are therapeutically promising targets for cancer patients. Cancer patients may benefit from anti-NETs therapy, especially when combined with immune checkpoint inhibitors.
